# Shortcomings of protocols of drug trials in relation to sponsorship as identified by Research Ethics Committees: analysis of comments raised during ethical review

**DOI:** 10.1186/1472-6939-15-83

**Published:** 2014-12-10

**Authors:** Marlies van Lent, Gerard A Rongen, Henk J Out

**Affiliations:** Clinical Research Centre Nijmegen, Department of Pharmacology – Toxicology, Radboud University Medical Center, Nijmegen, The Netherlands; Department of Pharmacology – Toxicology, Radboud University Medical Center, Nijmegen, The Netherlands; Department of Internal Medicine, Radboud University Medical Center, Nijmegen, The Netherlands; Teva Pharmaceuticals, Amsterdam, The Netherlands

**Keywords:** Research ethics committees, Ethical review, Research protocols, Drug trials, Sponsorship, Pharmaceutical industry, Shortcomings

## Abstract

**Background:**

Submission of study protocols to research ethics committees (RECs) constitutes one of the earliest stages at which planned trials are documented in detail. Previous studies have investigated the amendments requested from researchers by RECs, but the type of issues raised during REC review have not been compared by sponsor type. The objective of this study was to identify recurring shortcomings in protocols of drug trials based on REC comments and to assess whether these were more common among industry-sponsored or non-industry trials.

**Methods:**

Retrospective analysis of 226 protocols of drug trials approved in 2010–2011 by three RECs affiliated to academic medical centres in The Netherlands. For each protocol, information on sponsorship, number of participating centres, participating countries, study phase, registration status of the study drug, and type and number of subjects was retrieved. REC comments were extracted from decision letters sent to investigators after review and were classified using a predefined checklist that was based on legislation and guidelines on clinical drug research and previous literature.

**Results:**

Most protocols received comments regarding participant information and consent forms (n = 182, 80.5%), methodology and statistical analyses (n = 160, 70.8%), and supporting documentation, including trial agreements and certificates of insurance (n = 154, 68.1%). Of the submitted protocols, 122 (54.0%) were non-industry and 104 (46.0%) were industry-sponsored trials. Non-industry trials more often received comments on subject selection (n = 44, 36.1%) than industry-sponsored trials (n = 18, 17.3%; RR, 1.58; 95% CI, 1.01 to 2.47), and on methodology and statistical analyses (n = 95, 77.9% versus n = 65, 62.5%, respectively; RR, 1.18; 95% CI, 1.01 to 1.37). Non-industry trials less often received comments on supporting documentation (n = 72, 59.0%) than industry-sponsored trials (n = 82, 78.8%; RR, 0.83; 95% CI, 0.72 to 0.95).

**Conclusions:**

RECs identified important ethical and methodological shortcomings in protocols of both industry-sponsored and non-industry drug trials. Investigators, especially of non-industry trials, should better prepare their research protocols in order to facilitate the ethical review process.

## Background

Before new drugs can enter the market, they are extensively investigated in clinical trials. Also after registration, clinical drug research is important to improve our knowledge on safe and effective use of drugs [1]. In addition, drugs are frequently used as tools to investigate physiology and pathophysiology in humans in vivo. Prior to the recruitment of trial participants, research protocols need to be evaluated by research ethics committees (RECs) to assess whether all scientific, ethical, and legal requirements for conducting drug research with human subjects are met [2, 3]. Studies may raise questions of informed consent, adequate subject selection, scientific validity, acceptable risk-benefit ratios, and the value of the research to society in relation to the burdens to participants [4].

Submission of study protocols to RECs constitutes one of the earliest stages at which planned trials are documented in detail. Prior studies on ethical review of protocols involving human subjects have investigated the type of amendments and clarifications requested from researchers by RECs. For many protocols, some type of amendment was needed before approval [5–7]. RECs frequently commented on inadequacies in informed consent forms, possible risks to participants, methodological and statistical issues in protocols, and missing or incorrect supporting documentation [5–10]. However, several studies on REC review included small numbers of protocols [5, 9, 10], or focused on protocols submitted to single RECs so resulting findings may be specific to the committee studied [5, 6]. In addition, although the issues raised by RECs may depend on the intervention tested, previous studies have included protocols regardless of the type of intervention [5–8, 10].

The type and extent of shortcomings detected by RECs may also be related to sponsorship of trials. Pharmaceutical companies generally have larger budgets and a better research infrastructure compared to non-profit organizations, which could lead to higher protocol quality. However, it has also been suggested that the pharmaceutical industry’s pursuit of profit and favourable study results may have negative effects on research quality [11, 12]. Studies based on published trials have indicated that methodological quality is comparable for industry-sponsored and non-industry trials [12–14]. However, for a cohort of trials submitted to a Dutch REC, it was shown that non-industry trials have more problems in recruiting the required number of subjects than studies initiated by pharmaceutical companies [15]. This was confirmed in a study with protocols approved by RECs in Switzerland, Germany, and Canada indicating that industry-sponsorship was associated with lower rates of trial discontinuation due to poor recruitment [16]. The shortcomings of protocols that are identified during REC review have not been compared by sponsor type though.

The objective of this explorative study was to identify recurring shortcomings in protocols of drug trials and to assess whether these are more common among industry-sponsored or non-industry trials. Protocols submitted to three RECs affiliated to academic medical centres in The Netherlands were included and REC decision letters sent to researchers after review were accessed. REC comments were extracted and classified using a predefined checklist that was based on legislation and guidelines on clinical drug research and previous literature. It was examined whether there was a relationship between the occurrence of certain types of comments and industry sponsorship.

## Methods

### Selection of RECs and protocols

All eight RECs affiliated to academic medical centres in The Netherlands were asked to provide access to submitted protocols and their correspondence with investigators. These eight committees together review 70% of all medical research with human subjects conducted in The Netherlands [17]. RECs located in Nijmegen (CMO), Amsterdam (METc VUmc) and Leiden (CME) agreed to participate. Other committees did not participate due to logistical or confidentiality issues. All protocols approved in 2010–2011 were included, if they were classified as trials with medicinal products.

### Extraction of trial characteristics

For each protocol, information was retrieved on sponsor type, number of participating centres, participating countries, study phase, type of subjects, anticipated number of subjects, and whether the study drug was registered in The Netherlands. Data were extracted from a standardized form used in The Netherlands to prospectively document characteristics of planned trials (ABR form). Trials were classified as non-industry or industry-sponsored trials. For industry-sponsored trials, a pharmaceutical company was explicitly reported as the study sponsor in the ABR form. We verified whether the company indeed had the overall responsibility for the conduct of the trial using the trial protocol, clinical trial agreement and correspondence between the REC and investigators. Non-industry trials were investigator-initiated studies for which no associations with pharmaceutical companies were reported, or investigator-initiated studies that received study medication or financial support from manufacturers which did not have any responsibility for the content of the protocol.

### Extraction and classification of REC comments

For the classification of REC comments, a comprehensive checklist was composed prior to accessing REC decision letters (Table 1). The content of this checklist was based on national and international legislation and guidelines on the conduct of drug research with human subjects, and previous literature on this topic [2–4, 18–21]. This resulted in a checklist consisting of thirteen review criteria, that need to be considered by RECs to assess whether all scientific, ethical, and legal requirements for drug trials are met. For each trial, the issues raised by RECs were extracted from REC decision letters that were sent to investigators after protocol review. As these comments were not described in a standardized manner in REC letters, all data were first fully transcribed and subsequently categorized using the classification checklist. During the extraction of REC comments, there was no blinding for sponsorship as this was unfeasible due to logistical reasons. In this explorative study, the categorization of comments was performed by one author. Submitted protocols could be discussed by RECs at several occasions before receiving approval. All correspondence between RECs and investigators prior to approval was included in this study. Furthermore, the number of days between protocol submission and final REC approval was determined, which consisted of time attributable to RECs and time used by researchers to respond to comments.

**Table 1:** **Classification checklist for REC review of protocols of drug trials**

Review criteria	Definition
**1. Proportionality**	Risks and burdens to subjects are reasonable in relation to anticipated benefits
	Trial leads to important medical knowledge and/or considerable health benefits
	Participation does not involve unacceptable/disproportionate risks or burdens to subjects, use of placebo in trial is ethically justified
**2. Minimisation of risks & burdens**	Risks and burdens to subjects are minimized
	Research question cannot be answered without inclusion of human subjects
	Research question cannot be answered by more simple or less riskful/burdensome research
**3. Privacy & confidentiality**	Adequate provisions are made to protect privacy of subjects and maintain confidentiality of data and body tissues
	Trial data and body tissues are adequately coded, stored and protected, with restricted access for third parties
**4. Patient safety**	Adequate provisions are made to ensure safety of subjects during the trial
	Collected data are adequately monitored and (if necessary) a Data and Safety Monitoring Board (DSMB) is installed
	Individual and group level stopping criteria are adequate
**5. Recruitment process**	Recruitment methods and payments to subjects are acceptable
	Investigator has access to population that allows recruitment of required number of subjects
	Subjects are given adequate opportunity and time to ask questions and decide about participation
**6. Information sheet & consent form**	Subject information sheet & consent form contain all required elements
	The length, structure and language use of the information sheet & consent form will allow subjects to understand them correctly
**7. Subject selection**	Subject selection is appropriate to answer the research question
	Inclusion and exclusion criteria are adequate and complete, equity in subject selection
**8. Protection vulnerable subjects**	When vulnerable subjects are included, there are additional safeguards to protect their rights and well-being
	No vulnerable subjects are included, unless they may benefit themselves from participation or trial cannot be conducted without them
**9. Methodology & statistical analysis**	Trial design/methodology is appropriate and properly motivated in protocol
	Selected primary & secondary endpoints and dosage regimen are appropriate
	Sample size calculations and planned statistical analyses are adequate
**10. Product information**	Product information on the medicinal product(s) used in the study is adequate
	Investigator’s Brochure (IB) and Investigational Medicinal Product Dossier (IMPD)/ Summary of Product Characteristics (SPC) are acceptable
**11. Supporting documentation**	Other documents submitted as part of the research proposal are acceptable
	Data in ABR form are complete and correct and the Clinical Trial Agreement is in accord with Dutch regulations
	Required insurances for medical research with human subjects are arranged
**12. Facilities & research staff**	Research staff members are experienced and qualified to conduct trial procedures
	Investigator has adequate facilities to conduct the trial
**13. Financial aspects**	All costs incurring during the trial are adequately covered
	Compensation fees paid to investigators or institutions are proportional to the size, nature, and purpose of the trial
	Conditions (e.g. financial interests) leading to conflicts of interest are prevented

### Statistical analysis

The association between sponsor type and the number of trials that received comments after REC review was analyzed using generalized linear models (log-binomial) for each of the thirteen review criteria. We controlled for the REC that reviewed the protocol by including both sponsor type and REC as predictors in the model, and tested for interaction between sponsor type and REC. Associations between sponsor type and the number of trials that received comments were estimated with relative risks (RRs) and 95% confidence intervals (CIs). The total time between protocol submission and REC approval was compared by sponsor type and REC using a two-way ANOVA. We tested for interaction between sponsor type and REC. As time to approval was positively skewed, data were Ln-transformed before testing for differences between groups. P < .05 was considered statistically significant. Because of the explorative character of this study, P-values were not adjusted for multiple comparisons. Statistical analyses were performed using SPSS software (version 20; Chicago, Illinois).

### Ethics

To assure confidentiality of information in submitted protocols and correspondence between RECs and investigators, confidentiality agreements were signed before gaining access to the data. As standard REC procedures were unchanged, investigators were not informed about this study. Formal REC approval was not required for this study as no human subjects were involved.

## Results

In 2010 and 2011, 226 protocols of drug trials were approved by three Dutch RECs (Nijmegen, n = 74; Amsterdam, n = 76; Leiden, n = 76) (Table 2). In 2010, 123 (54.4%) protocols were approved, compared to 103 (45.6%) in 2011. Of the submitted trials, 104 (46.0%) were sponsored by the pharmaceutical industry and 122 (54.0%) were non-industry trials, of which 31 were financially or materially supported by a for-profit organisation (13.7% of the total number of trials).

**Table 2:** **Characteristics of drug trials approved in 2010-2011**

	Protocols
**Total, n (%)**	226 (100)
**REC**	
Nijmegen	74 (32.7)
Amsterdam	76 (33.6)
Leiden	76 (33.6)
**Year of approval**	
2010	123 (54.4)
2011	103 (45.6)
**Sponsor type**	
Non-industry	91 (40.3)
Non-industry, supported by industry	31 (13.7)
Industry-sponsored	104 (46.0)
**Number of centres**	
Single centre	100 (44.2)
Multicentre	126 (55.8)
**NL only or international trial**	
NL trial	131 (58.0)
International trial	95 (42.0)
**Phase**	
Phase 1	21 (9.3)
Phase 2	62 (27.4)
Phase 3	62 (27.4)
Phase 4	17 (7.5)
Other	64 (28.3)
**Study drug registered in NL**	
Yes, for the same indication/dosage as studied in protocol	50 (22.1)
Yes, for different indication/dosage than studied in protocol	74 (32.7)
No	79 (35.0)
Not reported whether drug was registered in NL	23 (10.2)
**Type of subjects**	
Adult subjects capable of giving informed consent	208 (92.0)
Adult subjects not capable of giving informed consent and/or minors <18 years	7 (3.1)
Adult subjects capable of giving informed consent and adults not capable of giving informed consent or minors <18 years	11 (4.9)
**Anticipated number of subjects, median (IQR)**	
Number of subjects (total)	59 (24–240)
Number of subjects (NL trials)	34 (20–60)
Number of subjects (international trials)	300 (120–800)

Most studies were multicentre trials (n = 126; 55.8%) and were mostly conducted in The Netherlands only (n = 131; 58.0%) (Table 2). 31 of the 126 multicentre studies (24.6%) were performed in The Netherlands only, whereas 95 (75.4%) were multinational multicentre studies. Trials were categorized as phase 1 (n = 21; 9.3%), phase 2 (n = 62; 27.4%), phase 3 (n = 62; 27.4%), phase 4 (n = 17; 7.5%), or other trials involving medicinal products (n = 64; 28.3%). In 50 trials (22.1%), a drug registered in The Netherlands for the same indication/dosage as studied in the trial protocol was used, while 74 (32.7%) used drugs that were registered for a different indication/dosage than studied in the protocol, and 79 (35.0%) used drugs that were not registered in the Netherlands. For 23 trials (10.2%), it was not reported whether the study drug was registered. Most trials recruited adult subjects capable of giving informed consent (n = 208; 92.0%), while 7 (3.1%) trials included adult subjects not capable of giving informed consent and/or minors <18 years, and 11 (4.9%) included both types of subjects. The median number of subjects per trial was 59 (interquartile range (IQR), 24–240). The median number of subjects in trials conducted in The Netherlands only was 34 (IQR, 20–60), compared to 300 (IQR, 120–800) in multinational trials.

In Table 3, random examples of comments raised during REC review are presented to illustrate each type of shortcoming. Table 4 shows the number of trials that received comments for each of the review criteria. Most trials received comments on the subject information sheet and consent form (n = 182, 80.5%), methodology and planned statistical analyses (n = 160, 70.8%), and supporting documentation, including clinical trial agreements and certificates of insurance (n = 154, 68.1%). In contrast, RECs rarely commented on the minimization of risks and burdens to subjects (n = 26, 11.5%), financial aspects (n = 39, 17.3%), and the protection of vulnerable subjects (n = 9, 4.0%).

**Table 3:** **Examples of REC comments on protocols of drug trials**

Review criteria	Example
**1. Proportionality**	*“The committee has serious doubts regarding the proportionality of the study, because the trial is very burdensome for participants and a number of potentially risky interventions are being combined”.*
	*“The committee would like to hear the expected benefit(s) of the study drug from the investigators; the clinical relevance of the trial is not clear”.*
**2. Minimisation of risks & burdens**	*“The committee wonders why it is necessary to burden patients with completion of the proposed quality of life questionnaires; much is already known about the quality of life of this group of patients”.*
	*“The investigators need to convince the committee of the usefulness of repeating skin biopsies and synovial biopsies”.*
**3. Privacy & confidentiality**	*“The committee notes that questionnaires should be stored coded; there should be no personal data on the questionnaires, as is the case now for a number of questionnaires”.*
	*“Trial medication is home delivered to participants by courier; the committee wants to know which provisions are made to prevent that participant addresses are known by the manufacturer”.*
**4. Patient safety**	*“The committee wonders why no individual and group level stopping rules are defined and no DSMB is installed for this trial”.*
	*“The committee wonders whether it is possible, in view of patient safety, to sequentially include participants in this trial”.*
**5. Recruitment process**	*“The minimal time of 24 h to decide about participation is too short considering the chance of placebo treatment, and the potential risks and burdens of participating; it should be minimally 5 days”.*
	*“The committee is concerned whether enough patients can be included; this type of cancer is particularly seen in patients with metastases, but these patients cannot participate in the trial”.*
**6. Information sheet & consent form**	*“The written subject information is too long and too difficult (regarding both vocabulary and style); the committee would like to see a completely rewritten version, understandable to a layperson”.*
	*“It should be more clearly explained to which extent the trial treatment deviates from the standard treatment and what subjects must undergo additionally by participating in the trial”.*
**7. Subject selection**	*“The committee asks the investigators why they do not select subjects at inclusion based on pain complaints, while this is an objective of the trial”.*
	*“The committee has the impression that the exclusion criteria are not complete, considering the SPC text; in particular, the committee believes that children with heart disease or kidney failure should be excluded”.*
**8. Protection vulnerable subjects**	*“The committee would like to hear substantive arguments for the need to include minors; the investigators have not justified why this group should be involved in this trial”.*
	*“The committee would like to hear how subjects and their relatives are informed on the procedures in case of resistance by subjects during the trial”.*
**9. Methodology & statistical analysis**	*“The sample size calculation does not reflect the primary analysis described as a (mixed effect) repeated measures ANOVA; the current calculation does not reflect that there are 10 VAS pain measurements”.*
	*“The trial is designed as cross-over; because of potential carry-over effects, the committee believes that a parallel comparison of 2 groups should be preferred”.*
**10. Product information**	*“The committee needs more information on the endothelin receptor antagonists; information on product characteristics, pharmacological characteristics, efficacy and safety, and pharmacokinetics is missing”.*
	*“The data in the IMPD are from 1997; the committee wants to know whether this batch is still being used or whether there has been a more recent production”.*
**11. Supporting documentation**	*“The text in the Clinical Trial Agreement on (premature) termination of the trial and publication of results does not correspond to provisions in the Dutch regulation on assessment of Clinical Trial Agreements”.*
	*“The committee wants to know whether the sponsor takes responsibility for the clinical trial liability insurance for all participating centres”.*
**12. Facilities & research staff**	*“A student cannot be principal investigator for this trial, because a student lacks experience and cannot take final responsibility for the trial”.*
	*“The committee believes that, considering the treatment during the trial, an oncologist should be involved in the design and conduct of the trial”.*
**13. Financial aspects**	*“The committee would like to see an exact justification of the 100.000 euro that the department will receive for conducting the trial”.*
	*“The committee does not agree with the independent physician appointed for 3 of the participating centres; this person also works as a physician for the pharmaceutical company sponsoring this trial”.*

**Table 4:** **Analysis of REC comments - non-industry (n = 122) vs industry-sponsored trials (n = 104)**

Review criteria	Protocols with comments, n (%)	Relative risk (95% CI)*	P-value
**1. Proportionality**	61 (27.0)		
Non-industry	34 (27.9)	0.95 (0.62-1.45)	.805
Industry	27 (26.0)		
**2. Minimisation of risks & burdens**	26 (11.5)		
Non-industry	15 (12.3)	0.92 (0.44-1.91)	.820
Industry	11 (10.6)		
**3. Privacy & confidentiality**	63 (27.9)		
Non-industry	29 (23.8)	0.93 (0.65-1.34)	.695
Industry	34 (32.7)		
**4. Patient safety**	61 (27.0)		
Non-industry	36 (29.5)	1.31 (0.84-2.04)	.228
Industry	25 (24.0)		
**5. Recruitment process**	47 (20.8)		
Non-industry	30 (24.6)	1.05 (0.63-1.73)	.866
Industry	17 (16.3)		
**6. Information sheet & consent form**	182 (80.5)		
Non-industry	100 (82.0)	1.04 (0.94-1.13)	.468
Industry	82 (78.8)		
**7. Subject selection**	62 (27.4)		
Non-industry	44 (36.1)	1.58 (1.01-2.47)	.045
Industry	18 (17.3)		
**8. Protection vulnerable subjects**	9 (4.0)		
Non-industry	6 (4.9)	1.53 (0.38-6.07)	.549
Industry	3 (2.9)		
**9. Methodology & statistical analysis**	160 (70.8)		
Non-industry	95 (77.9)	1.18 (1.01-1.37)	.038
Industry	65 (62.5)		
**10. Product information**	65 (28.8)		
Non-industry	38 (31.1)	1.16 (0.75-1.78)	.507
Industry	27 (26.0)		
**11. Supporting documentation**	154 (68.1)		
Non-industry	72 (59.0)	0.83 (0.72-0.95)	.006
Industry	82 (78.8)		
**12. Facilities & research staff**	57 (25.2)		
Non-industry	39 (32.0)	1.60 (0.98-2.60)	.060
Industry	18 (17.3)		
**13. Financial aspects**	39 (17.3)		
Non-industry	25 (20.5)	1.65 (0.90-3.01)	.103
Industry	14 (13.5)		

* Relative risks are controlled for the REC that reviewed the protocol.

Non-industry trials more often received comments on subject selection (n = 44, 36.1%) than industry-sponsored trials (n = 18, 17.3%; RR, 1.58; 95% CI, 1.01 to 2.47) (Table 4). More non-industry trials received comments on methodology and statistical analyses (n = 95, 77.9%) compared to industry-sponsored trials (n = 65, 62.5%; RR, 1.18; 95% CI, 1.01 to 1.37). In contrast, non-industry trials less often received comments on supporting documentation (n = 72, 59.0%) than industry-sponsored trials (n = 82, 78.8%; RR, 0.83; 95% CI, 0.72 to 0.95). There was a non-significant trend indicating that non-industry trials more often received comments related to facilities and research staff (n = 39, 32.0%) compared to industry-sponsored trials (n = 18, 17.3%; RR, 1.60; 95% CI, 0.98 to 2.60). The proportion of trials that received comments was comparable between RECs for protection of vulnerable subjects and product information. For the other review criteria, the proportion of trials with comments was significantly different between RECs (data not shown). For product information and supporting documentation, the interaction between sponsor type and REC was p < .05. However, RRs for the association between sponsorship and these review criteria were comparable for the individual RECs and the interaction was considered not relevant with regard to the effect of sponsorship.

The median time to protocol approval was 112 days (IQR, 78–163) (Table 5). Time to approval was not significantly different between industry-sponsored (119 days; IQR, 84–169) and non-industry trials (101 days; IQR, 75–158, p = .298). Time to approval was longer for protocols submitted in Nijmegen (148 days; IQR, 89–203) than for those submitted in Amsterdam (113 days; IQR 87–150) or Leiden (91 days; IQR, 65–127, overall p = .000). There was no interaction between sponsor type and REC regarding time to approval.

**Table 5:** **Time to approval of submitted protocols**

	Time to approval in days, median (IQR)	P-value
**Total protocols (n = 226)**	112 (78–163)	
**Sponsor type**		.298
Non-industry (n = 122)	101 (75–158)	
Industry-sponsored (n = 104)	119 (84–169)	
**REC**		.000
Nijmegen (n = 74)	148 (89–203)	
Amsterdam (n = 76)	113 (87–150)	
Leiden (n = 76)	91 (65–127)	
**Nijmegen vs Amsterdam**		.026
**Nijmegen vs Leiden**		.000
**Amsterdam vs Leiden**		.001

## Discussion

In this study, recurring shortcomings in protocols of drug trials were identified based on REC comments and it was assessed whether these were more common among industry-sponsored or non-industry trials. Most protocols received comments regarding informed consent forms, methodology and planned statistical analyses, and supporting documentation including clinical trial agreements and certificates of insurance. This corresponds to findings of previous studies on the issues raised by RECs [5–7, 9]. In addition, a recent evaluation of the Dutch law on medical research with human subjects also indicated that RECs give a lot of attention to improvement of participant information [22]. RECs rarely addressed the minimization of risks and burdens to subjects, financial aspects, and the protection of vulnerable subjects. The last finding is hardly surprising, as 90% of the protocols included adult subjects capable of giving informed consent.

Non-industry protocols more often received comments related to methodology and statistical analyses and the selection of participants than industry-sponsored protocols, while research based on published trials indicated that methodological quality was comparable for industry-sponsored and non-industry trials [12–14]. RECs may play an important role in improving the quality of submitted protocols, which could explain the observed absence of substantial differences in methodological quality in relation to sponsorship among published trials. In contrast, industry-sponsored trials more often received comments on supporting documentation submitted alongside protocols. This difference seemed to be associated with REC comments on clauses in clinical trial agreements for industry-sponsored trials, in particular regarding the publication of results and premature termination of trials.

The time to protocol approval was comparable for non-industry and industry-sponsored trials, but was significantly different between RECs. Time to approval may be related to the quality and characteristics of submitted protocols, but may also reflect the efficiency of the review process. The REC in Nijmegen received lower proportions of industry-sponsored studies and trials with drugs not registered in The Netherlands. There may be other differences between submitted protocols that were not detected in this study. In Leiden and Amsterdam, protocols are reviewed by science committees before submission to the REC, which may reduce the time needed by RECs to review methodological aspects. In addition, investigators submitting protocols to the REC in Leiden are invited to discuss their study with a pre-advisor prior to REC meetings. In The Netherlands, RECs should complete reviews of drug trials within sixty days [18]. The clock stops when RECs ask questions to investigators, and restarts when the required information is provided. However, as time to approval consisted of time attributable to RECs and time used by researchers to respond to comments, no definite conclusions can be drawn.

Due to the retrospective design of this study, included protocols were already reviewed before RECs were asked to participate in this study and the behaviour of REC members was not influenced by awareness of an ongoing investigation. As we had unrestricted access to submitted protocols and correspondence between RECs and researchers, we were able to extract trial characteristics and REC comments without having to obtain permission from researchers or rely on a survey of researchers to obtain these data, excluding the introduction of response bias [23]. Although there was no response bias with respect to individual studies, there may be a response bias in terms of RECs if the committees that declined to take part somehow differed from participating committees. Nevertheless, by inclusion of protocols submitted to three RECs that are among the eight largest committees in The Netherlands regarding the number of studies annually reviewed, more than 20% of all drugs trials approved in The Netherlands in 2010–2011 were evaluated.

Many studies have focused on the methodological quality of published trials [12–14] or followed protocols approved by RECs until publication [23, 24]. In contrast, we identified shortcomings of protocols based on REC comments raised prior to trial initiation. Quality assessments in previous studies may have been affected by incomplete reporting in published articles, as evaluations of the risk of bias in trials may have been confused with assessment of the adequacy of reporting [25]. REC comments may not inherently provide an objective reflection of the quality of submitted protocols, as it has been shown that REC review is variable regarding the decisions made on protocols [26–28]. Comments that are raised may be influenced by local variation in requirements, and may vary by REC even for the same protocol with multicenter trials [28, 29]. In this study, the proportion of protocols that received comments varied by REC for most of the review criteria. It is difficult to determine whether this reflects variable protocol quality or variable review by RECs.

This study has certain limitations. First, we focused on protocols submitted to Dutch RECs. In addition to the variability found between RECs within the same country, ethical review processes also vary between countries [30, 31]. This limits the generalizability of our findings to non-Dutch RECs, although 40% of the included trials were conducted in multiple countries. This study included trials that eventually received a favourable opinion. We did not evaluate protocols that were rejected by RECs. Although only 3% of all trials annually reviewed in The Netherlands are rejected [17], it may be interesting to consider this group as the chance of rejection might be associated with sponsorship. However, this was beyond the scope of the current study.

In this explorative study, the categorization of REC comments was performed by one author, while this would ideally have been done by two or more independent assessors and disagreements resolved by consensus. The categorisation of REC comments may be considered as subjective. However, to our knowledge, this is the first study in which shortcomings of protocols of drug trials as identified by RECs have been compared by sponsor type. The internal and external validity of the classification checklist used in this study could be further verified in future studies with different assessors and different samples of research protocols submitted to RECs.

Finally, industry-sponsored and non-industry trials may not be similarly addressed by RECs. Pharmaceutical companies commonly perform large multinational trials, while non-industry trials are more often initiated and conducted by local investigators. In this study, 90% of the industry-sponsored trials were multicenter studies, compared to 30% of the non-industry trials. RECs that are involved at a single site of a large multicenter trial might feel constrained in their ability to modify the trial design and limited in what changes they can make to informed consent forms. In addition, multinational industry trials may have already implemented comments from RECs in other countries before submission to Dutch RECs.

## Conclusions

In conclusion, RECs identified important ethical and methodological shortcomings in protocols of both industry-sponsored and non-industry drug trials. Investigators, especially of non-industry trials, should better prepare their research protocols in order to facilitate the ethical review process.

## Abbreviations

CI: Confidence interval
IQR: Interquartile range
REC: Research ethics committee
RR: Relative risk.
